# Unilateral mandibular condylar hyperplasia with ipsilateral masticator muscle and parotid gland hypertrophy: a rare incidental finding on brain MRI

**DOI:** 10.1186/s12891-025-09263-x

**Published:** 2025-11-07

**Authors:** Saburi Singhania, Pratapsingh Parihar, Shivali Kashikar, Nirja Thakere, Umang Jajoo, Komal Rathod

**Affiliations:** Department of Radiodiagnosis Jawaharlal Nehru Medical College (JNMC), Datta Meghe Institute of Higher Education and Research (DMIHER), Sawangi, Wardha, 442001 Maharashtra India

**Keywords:** Mandibular condylar hyperplasia, Adolescent, Facial asymmetry, MRI, Volumetric analysis

## Abstract

**Background:**

Mandibular condylar hyperplasia (MCH) is a rare developmental disorder characterized by excessive growth of the mandibular condyle, leading to facial asymmetry, functional impairment, and dental malocclusion. Early radiological identification is essential for timely intervention, particularly when parotid gland involvement masks the underlying skeletal pathology.

**Case presentation:**

A 15-year-old male presented with an 8–9 month history of painless left parotid swelling. MRI revealed a unique triad comprising a bulky left parotid gland measuring 4.3 × 3.2 cm compared to 3.1 × 2.4 cm on the right, left masseter hypertrophy with a volume of 28.3 cm³ versus 18.7 cm³ on the right representing a 51% increase, and left medial pterygoid hypertrophy measuring 15.2 cm³ versus 10.1 cm³ on the right showing a 50% increase. Quantitative analysis demonstrated significant left condylar enlargement with 31% height asymmetry, measuring 24.3 mm on the left versus 18.5 mm on the right, and increased anteroposterior diameter of 11.2 mm versus 8.3 mm. SPECT imaging was performed to assess growth activity, guiding subsequent management decisions between immediate surgical intervention versus conservative monitoring.

**Conclusion:**

This case demonstrates how unilateral condylar hyperplasia can present primarily as parotid swelling, potentially misdirecting clinical attention. Quantitative volumetric analysis proved essential for diagnosis. The documented 31% condylar asymmetry with preserved function illustrates effective neuromuscular compensation, providing a 6–12 month window for intervention before symptom development. Early multidisciplinary collaboration is crucial for optimal outcomes.

## Introduction

This case report presents a unique triad of unilateral condylar hyperplasia (UCH), masticator muscle hypertrophy, and secondary parotid gland enlargement in an adolescent with preserved function despite significant anatomical changes—a presentation rarely documented in the literature. The novelty lies in demonstrating how UCH can misleadingly present as parotid pathology, potentially delaying correct diagnosis and treatment.

UCH is an uncommon developmental disorder resulting in asymmetrical mandibular overgrowth, typically manifesting during adolescence [1–3]. While the condition’s pathogenesis involves hyperactive condylar growth centers leading to persistent endochondral ossification [4, 5], its clinical presentation can be subtle, often mimicking soft tissue pathologies [2, 6].

Current imaging modalities play complementary roles in UCH evaluation. MRI excels at soft tissue evaluation and volumetric assessment, while CT and CBCT provide optimal bone morphology visualization [7–10]. SPECT determines growth activity crucial for treatment planning, with a difference of 10% or greater in uptake or a 55:45 ratio indicating active growth [11–13]. Recent advances in surgical planning, including virtual surgical protocols and custom 3D-printed guides, have improved precision in condylectomy procedures and optimize surgical outcomes [14].

This case highlights the diagnostic value of quantitative multimodal imaging in identifying this rare triad and emphasizes the importance of recognizing secondary soft tissue changes that may obscure the underlying skeletal pathology. Early identification during adolescence represents a critical window for intervention, preventing long-term deformity and need for extensive reconstruction [1, 2, 15].

## Case presentation

A 15-year-old male presented with an 8–9 month history of painless left parotid swelling. Past medical history included self-limiting mumps one year prior, with no residual complications. Clinical examination revealed mild facial asymmetry with fullness over the left mandibular angle. The swelling was firm, non-tender, and not fixed to overlying skin. Occlusion was preserved with normal mouth opening of 42 mm interincisal distance and no TMJ dysfunction.

**Fig. 1:**
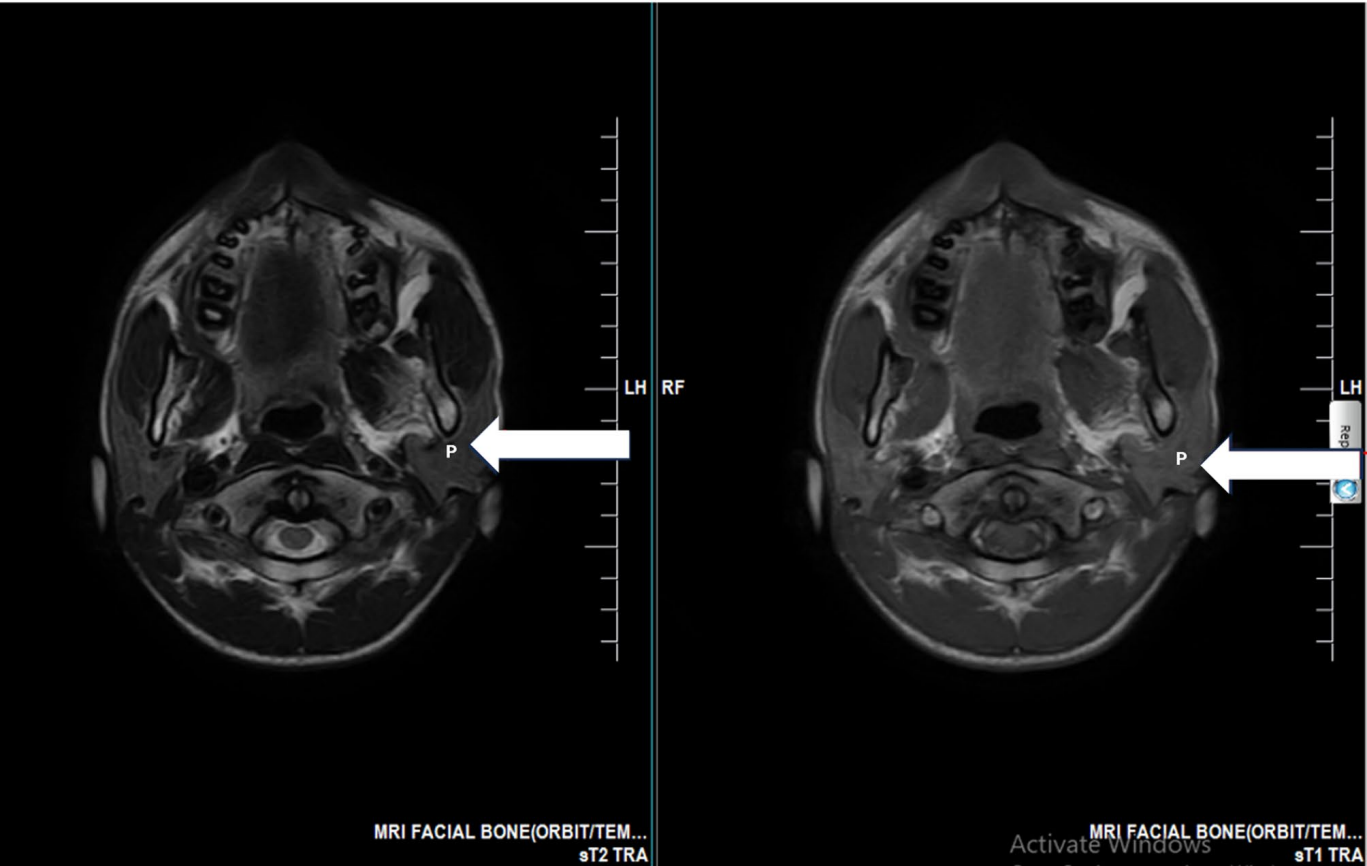
Axial MRI showing unilateral parotid gland enlargement. T2-weighted (left) and T1-weighted (right) axial images demonstrating a bulky and enlarged left parotid gland (white arrowhead, *P* = Parotid) compared to the normal-sized right parotid gland

Ultrasonography demonstrated diffuse left parotid enlargement measuring 4.3 × 3.2 cm compared to 3.1 × 2.4 cm on the right, with homogeneous echotexture suggesting reactive hypertrophy rather than primary pathology (Fig. 1). Panoramic radiography revealed vertical discrepancy in mandibular rami height with left-sided elongation. Due to inherent limitations of panoramic imaging including horizontal distortion, vertical magnification, and superimposition of structures [16, 17], advanced cross-sectional imaging was obtained.

**Fig. 2:**
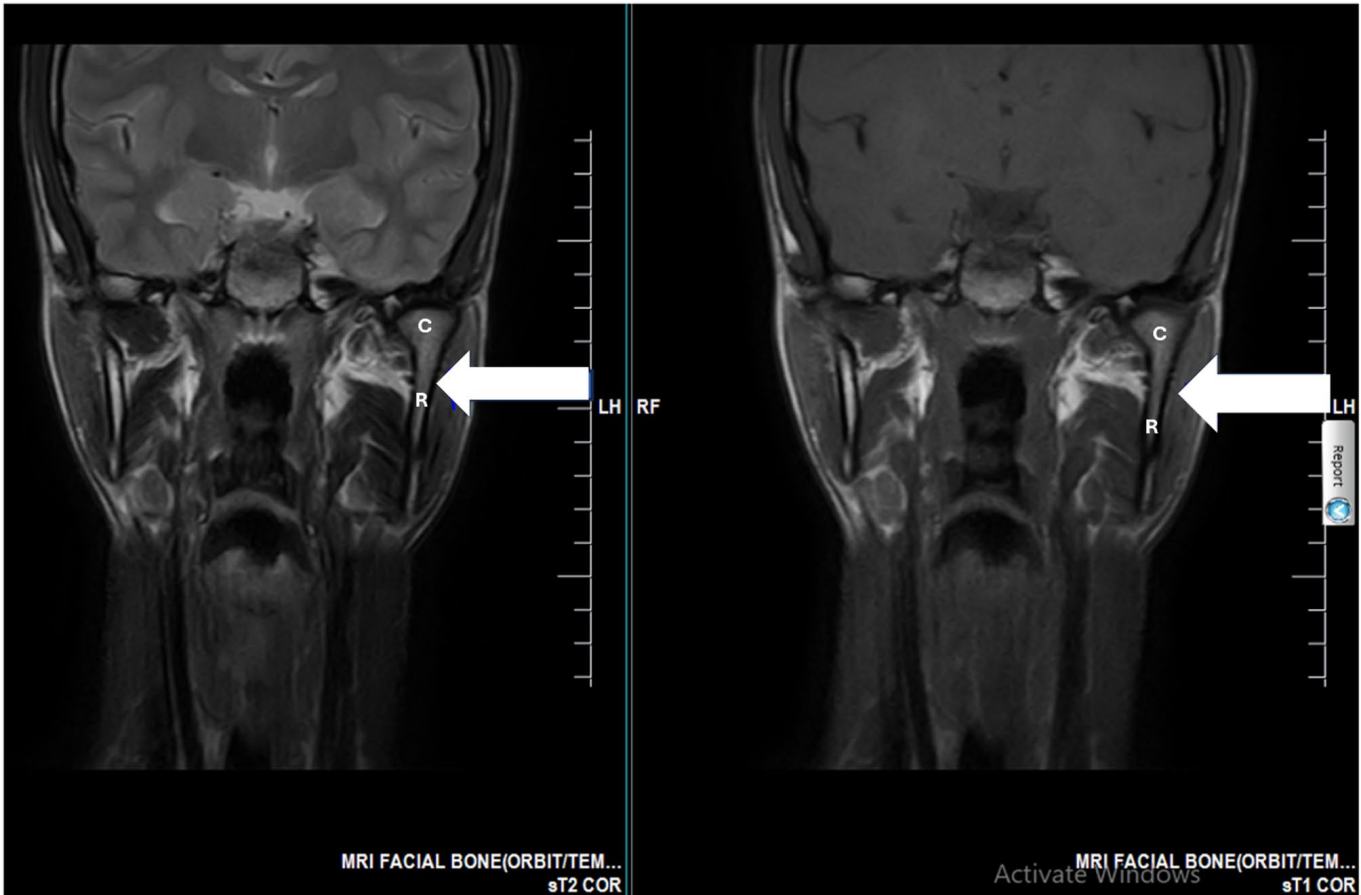
Coronal MRI showing left unilateral mandibular condylar hyperplasia. T2-weighted (Left) and T1-weighted (Right) coronal images showing asymmetrical thickening and elongation of the ramus and condyle of the mandible on the left side (white arrowhead, C = Condyle, R = Ramus) as the primary underlying pathology, when compared to the normal right side

**Fig. 3:**
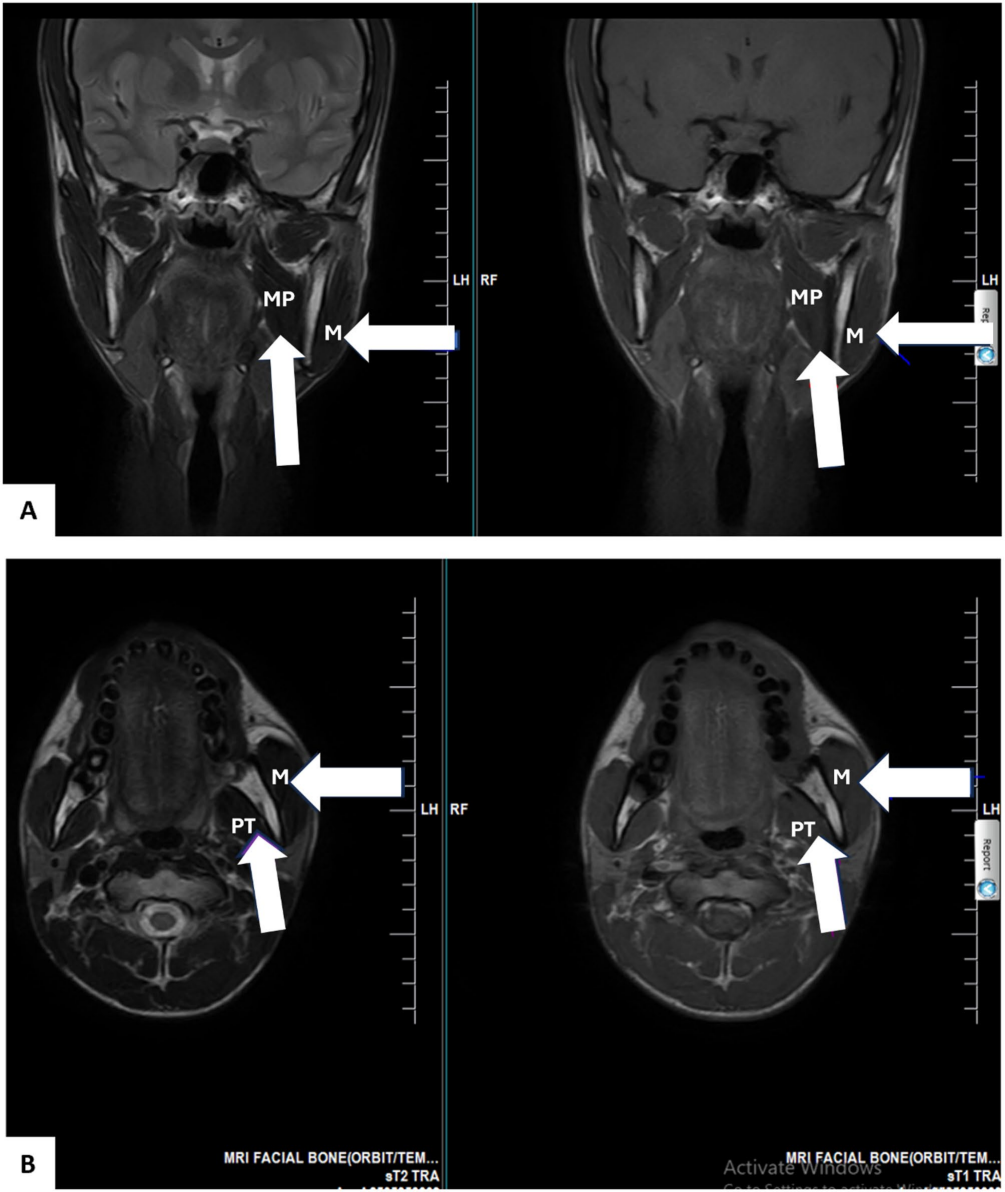
Muscular hypertrophy secondary to mandibular asymmetry. **A** Coronal T2-weighted (Left) and T1-weighted (Right) images showing significant hypertrophy of the left masseter muscle (white arrowhead, M = Masseter) and left medial pterygoid muscle (white arrow, MP = Medial Pterygoid). **B** Axial T2-weighted (Left) and T1-weighted (Right) images showing the increased bulk of the left masseter muscle (white arrowhead, M = Masseter) and left pterygoid muscles (white arrow, PT = Pterygoid) when compared to the right side

MRI quantitative analysis revealed significant asymmetries across multiple structures. The left condylar height measured 24.3 mm compared to 18.5 mm on the right, representing 31% asymmetry (Fig. 2). The left condylar anteroposterior diameter was 11.2 mm versus 8.3 mm on the right, showing 35% asymmetry, while the mediolateral width measured 19.8 mm on the left and 15.2 mm on the right, demonstrating 30% asymmetry. Volumetric analysis of the masticatory muscles showed left masseter volume of 28.3 cm³ compared to 18.7 cm³ on the right, indicating 51% hypertrophy, and left medial pterygoid volume of 15.2 cm³ versus 10.1 cm³ on the right, representing 50% hypertrophy (Fig. 3). The left ramus height measured 68.4 mm compared to 61.2 mm on the right, showing 11.8% elongation (Fig. 3). No joint effusion, disc displacement, or osseous pathology was identified. The preserved occlusion despite 31% condylar asymmetry indicated effective neuromuscular compensation.

### Management algorithm

Despite pending SPECT results at initial submission, we established a clear management protocol. If SPECT demonstrates active growth with greater than 10% uptake difference, high condylectomy would be performed within 3 months, accompanied by pre-operative orthodontic assessment and 6-month post-surgical follow-up. If SPECT shows inactive growth with less than 10% difference, conservative management with biannual monitoring would be pursued, considering orthodontic camouflage for asymmetry less than 5 mm and potential orthognathic surgery after skeletal maturity between ages 18–21.

Current management includes quarterly clinical assessments, serial photography for asymmetry progression monitoring, and multidisciplinary team coordination. SPECT imaging was completed 2 weeks post-submission to determine growth activity status.

## Discussion

### Clinical significance

This case demonstrates how unilateral condylar hyperplasia can present primarily as parotid swelling, potentially misdirecting clinical attention from the underlying skeletal pathology. The documented 31% condylar asymmetry with 50–51% secondary muscle hypertrophy represents a significant anatomical alteration that remained functionally compensated. This distinction has critical treatment implications, as addressing only the parotid enlargement without recognizing UCH would result in treatment failure and progressive deformity.

### Diagnostic approach and UCH classification

Our quantitative analysis confirmed hemimandibular hyperplasia based on Obwegeser-Makek criteria [18, 19]. The diagnosis was established by vertical elongation with a ramus height difference of 7.2 mm, three-dimensional condylar enlargement showing 31% asymmetry, preserved condylar morphology, and absence of horizontal mandibular elongation. This contrasts with hemimandibular elongation, which demonstrates horizontal mandibular body lengthening with normal condylar dimensions and chin deviation exceeding 5 mm [20].

The imaging hierarchy for UCH evaluation follows a systematic approach. MRI provides soft tissue volumetrics and TMJ disc assessment, while CBCT offers precise bone morphology when MRI suggests osseous pathology. SPECT determines growth activity, with greater than 10% uptake difference indicating active growth requiring surgical intervention [11–13]. Virtual surgical planning using MSCT data and specialized software has revolutionized treatment precision. Hegab et al. demonstrated that custom 3D-printed cutting guides based on virtual planning ensure accurate condylectomy execution, particularly crucial when inferior alveolar nerve transposition is required [14]. This comprehensive three-dimensional assessment optimizes outcomes and minimizes complications. Volumetric analysis guides the extent of condylectomy required, supporting our quantitative imaging approach.

### Differential diagnosis

Unilateral condylar hyperplasia was confirmed in this case based on 31% condylar height asymmetry with secondary muscle hypertrophy. Masseteric hypertrophy was excluded due to bilateral muscle involvement and medial pterygoid participation, which is unlikely in isolated MH [21]. Primary parotid pathology was ruled out by the homogeneous gland texture without focal lesions or ductal changes [9]. The possibility of an accessory parotid gland was considered, as recent anatomical studies have documented significant size variations in these structures. Ogut et al. (2025) reported accessory parotid glands ranging from 14.96 × 6.36 mm to 26.22 × 23.08 mm, located 8.9 mm from the zygomatic arch [22]. However, our case demonstrated diffuse enlargement of the main parotid gland rather than a discrete accessory structure, and the enlargement correlated with underlying skeletal asymmetry rather than representing an independent glandular anomaly. Chronic sialadenitis was excluded based on absence of inflammatory markers, ductal obstruction, or fibrosis. IgG4-related disease was considered unlikely given the unilateral presentation without systemic features [23, 24].

### Neuromuscular compensation and clinical implications

The preserved function despite 31% condylar asymmetry indicates effective compensation with important practical implications. This compensation provides a timing window of 6–12 months before symptom development, requiring quarterly monitoring assessments focusing on progressive mandibular deviation, occlusal interference development, and masticatory fatigue onset. The effective compensation predicts better post-surgical adaptation and indicates low immediate TMJ dysfunction risk. This compensation paradox, as described by Brionne et al. [25], explains why substantial morphological asymmetry may remain clinically silent, delaying intervention.

### Orthodontic and orthognathic considerations

Multidisciplinary planning depends on asymmetry severity. For mild discrepancies less than 5 mm, orthodontic camouflage alone using asymmetric mechanics with differential force application can achieve satisfactory results over 18–24 months. Moderate discrepancies between 5 and 10 mm require unilateral high condylectomy with pre-surgical orthodontic decompensation lasting 12–18 months followed by post-surgical refinement for 6–12 months. Severe asymmetry exceeding 10 mm necessitates bimaxillary osteotomy with possible distraction osteogenesis [26]. When distraction osteogenesis is employed for severe mandibular asymmetry, recent advances in bone healing enhancement show promise. Elhadidi et al. (2021) demonstrated that bone marrow aspirate concentrate application during mandibular distraction produced trends toward improved bone density (293 ± 100 HU vs. 176 ± 94 HU) and bone volume fraction (49.47% vs. 43.9%), though results did not reach statistical significance [27]. Patient-specific implants further enhance three-dimensional accuracy for orthognathic procedures [28], with comprehensive orthodontic-surgical protocols spanning 24–36 months total treatment time. Tsai et al. demonstrated successful camouflage treatment using asymmetric elastics and differential bracket systems, achieving satisfactory results in mild cases [29].

### Management strategy based on growth activity

Management decisions depend critically on SPECT findings [30]. Active growth with SPECT demonstrating greater than 10% difference mandates immediate surgical intervention to arrest progression. Recent evidence by Hegab et al. (2024) demonstrated excellent outcomes using virtual surgical planning with custom 3D-printed cutting guides in 16 patients with UCH [14]. Their treatment algorithm stratified patients based on technetium-99 m bone scan activity, with 31% of active UCH patients receiving proportional condylectomy with orthodontics alone, while 19% required condylectomy combined with orthognathic surgery. Virtual planning using MSCT data imported into Mimics software allowed precise quantification of condylectomy amount, with segmentation thresholds of 226–3071 HU adjusted for individual anatomy. Custom cutting guides ensured accurate intraoperative reproduction of the virtually planned resection, particularly important when inferior alveolar nerve transposition was required. Post-operative complications were limited to transient lip numbness in cases requiring inferior border osteotomy, which resolved within 6 months.

Conversely, inactive growth with SPECT showing less than 10% difference allows conservative approach with regular monitoring. In the Hegab series, 37.5% of inactive UCH patients chose orthodontics alone despite minimal asymmetry, while 12.5% underwent genioplasty with orthodontics for chin deviation correction. The decision for intervention in inactive cases depends on functional symptoms development, documented progressive asymmetry, or patient request for aesthetic correction.

### Study limitations

This single case report has inherent limitations. Long-term follow-up data and SPECT results were pending at submission. Histopathological confirmation was not obtained given the clear imaging diagnosis and conservative initial management. Future studies should include larger cohorts with complete volumetric analysis and long-term outcomes.

## Conclusion

This case highlights the diagnostic challenge when unilateral condylar hyperplasia presents primarily as parotid swelling. Quantitative volumetric analysis proved essential, revealing 31% condylar asymmetry with 50–51% secondary muscle hypertrophy. The preserved function despite significant anatomical changes demonstrates effective neuromuscular compensation, providing a critical intervention window. Early recognition through multimodal imaging and prompt multidisciplinary collaboration are essential to prevent progression and optimize outcomes in adolescent UCH patients.

## Data Availability

No datasets were generated or analysed during the current study.
